# Low Expression of FAM96B is Associated with Poor Prognosis in Hepatocellular Carcinoma

**DOI:** 10.5152/tjg.2024.24198

**Published:** 2024-12-16

**Authors:** Yuqun Tang, Liangke Tang, Haijian Du, Chaosheng Wu, Zhuangxiong Wang, Guanshui Luo, Ke He

**Affiliations:** 1 Cancer Minimally Invasive Therapies Centre, Guangdong Second Provincial General Hospital, Guangzhou, China; 2 Department of Thyroid and Breast Surgery, Southern Medical University, Hospital of Integrated Chinese and Western Medicine, Guangdong, China

**Keywords:** FAM96B, hepatocellular carcinoma, prognosis, immunohistochemistry

## Abstract

**Background/Aims:**

Recently, studies on FAM96B functions mainly focused on its role in maintaining the normal physiological function of cells. However, the clinical implications of FAM96B in hepatocellular carcinoma (HCC) are still unclear.

**Materials and Methods:**

FAM96B mRNA expression was detected in human HCC tissues and the matched nontumorous tissues by quantitative real-time reverse transcription (qRT-PCR) and then validated in The Cancer Genome Atlas (TCGA) database. Immunohistochemistry assay (IHC) was performed on all 137 cases of HCC samples to examine the protein level of FAM96B. Subsequently, the associations between FAM96B expression and clinicopathological parameters and prognosis were further analyzed.

**Results:**

The mRNA level of FAM96B was found to be significantly lower in HCC tissues compared to non-tumorous tissues, as observed in both the local hospital and TCGA database.Immunohistochemistry assay analysis revealed a decrease in FAM96B expression in 78 out of 137 cases, which was significantly associated with larger tumor size, higher Barcelona clinic liver cancer or Child stage, and early distant metastasis. Patients with low FAM96B levels tended to have an unfavorable disease-free and overall survival. Moreover, FAM96B was identified as an independent predictor of patient prognosis in both univariate and multivariate survival analyses. Mechanistically, FAM96B was found to inhibit cancer progression by inducing apoptosis in liver cancer cells and inhibiting their growth.

**Conclusion:**

Our findings provide the first evidence suggesting the involvement of FAM96B in the progression of HCC. Additionally, FAM96B could potentially serve as a marker for tumor recurrence and prognosis in HCC patients.

**Main Points**

- FAM96B expression in HCC tissues was downregulated.
- Low expression of FAM96B was correlated with advanced BCLC or Child stage, larger tumor size, and early distant metastasis.
- Patients with low FAM96B levels tended to have an unfavorable disease-free and overall survival.
- FAM96B was found to inhibit cancer progression by inducing apoptosis in liver cancer cells and inhibiting their growth.

## Introduction

Hepatocellular carcinoma (HCC) poses a significant challenge for treatment globally.^1^ It is classified as the sixth most prevalent form of cancer and the third leading contributor to mortality in cancer patients. In 2020, there were nearly 900 000 newly confirmed cases and 800 000 deaths from liver cancer worldwide. More seriously, the frequency of HCC cases is expected to see an upward trend in the forthcoming period.^1^ So far, there is no effective treatment for HCC. Common treatments include surgical resection,^2^ liver transplantation,^2^ radiofrequency ablation, and hepatic artery chemoembolization.^3^ Tyrosine kinase inhibitors, such as sorafenib,^4^ levotinib,^5^ and regglinide,^6^ have limited efficacy. Human HCC is generally believed to be closely related to hepatitis B virus (HBV) and hepatitis C virus (HCV) infection,^7^ aflatoxin B1, non-alcoholic fatty liver disease, cirrhosis, and long-term alcohol consumption.^8-10^ Development of HCC is related to many factors including the stimulation of oncogenes, the deactivation of genes with tumor-suppressive functions, and tumor metastasis suppressor genes.^11^ Given the limitations of traditional treatment for HCC, it is crucial to identify novel targets and prognosis predictors. This approach holds promise for treating cancers and facilitating precision medicine.

FAM96B (family with sequence similarity 96, member B) is located in 16q22.1-q22.3. It is a gene originally discovered by comparing the genomes of humans and nematodes based on the expression sequence tag (EST) database. However, so far, there is little study on the function of FAM96B both domestically and internationally. Previous studies showed that FAM96B could regulate VEGFR2 promoter activity by interacting with E2-2, a basic helix-loop-helix protein, thus fulfilling a crucial function in the growth, movement, and microtubule creation of vascular endothelial cells.^12^ In addition, Xiong et al^13^ found that FAM96B formed a physical interaction with prelamin A and might play a role in cell senescence. Recently, FAM96B was found to be crucial for DNA processing and is essential in maintaining DNA integrity through repair mechanisms, influencing genomic stability.^14^ Furthermore, FAM96B is a component of the MMXD complex (MMS19-FAM96B-XPD) and participates in the formation of the mitotic spindle, which is essential for the accurate segregation of chromosomes during cell division. Research showed that knockdown of FAM96B led to an increase in heteronuclear accumulation.^15^ According to research carried out by Stehling et al, findings have shown that FAM96B is pivotal in facilitating the assembly of the mitotic spindle by enlisting brain-type creatine kinase.^16^ Furthermore, it has been demonstrated to impede the aging of dental pulp stem cells by enhancing both their ability to differentiate into bone-forming cells and their rate of growth.^17^ However, research on the role of FAM96B in tumors is limited, with the exception of a report suggesting its potential to suppress breast cancer progression by regulating the Wnt/β-catenin signaling.^18^ Despite this, the expression levels and clinical implications of FAM96B in HCC have yet to be fully explored.

In this research, we conducted an investigation on the expression of FAM96B in 137 cases of HCC using an immunohistochemistry (IHC) assay. Moreover, we analyzed how the expression of FAM96B is related to clinicopathological characteristics and, ultimately, the postoperative survival rates of patients with HCC.

## Materials and Methods

### Clinical Information

In this investigation, matched tumorous samples and non-tumorous samples were collected from 137 HCC patients between May 2015 and August 2018 in the Department of Pathology, Guangdong Second Provincial General Hospital. The eligibility criteria for patients were as outlined: (1) HCC was confirmed by histopathologic diagnosis; (2) Preoperative treatment, including radiotherapy, chemotherapy, immunotherapy, and targeted therapy, was not used; (3) Complete clinical follow-up information was available; (4) Surgical resection was the treatment modality; (5) No history of other malignant tumors. Overall survival (OS) is the timeframe starting from liver cancer patients undergoing surgery to their eventual death. Disease-free survival (DFS) is the time span between the surgical procedure and the first occurrence of cancer recurrence or distant spread. Fresh tumor samples obtained after surgery were promptly treated with RNA later (Ambion, Austin, TX, USA) and kept frozen at −80°C. The Ethics Committee of Guangdong Second Provincial General Hospital has granted approval for this study (approval number: 2023-SZ-KY-008-04, date; December 20, 2023). All patients or their family members provided written informed consent, in compliance with the Helsinki Declaration.

### RNA Extraction and Quantitative Real-Time Reverse Transcription Polymerase Chain Reaction

RNA extraction and quantitative real-time reverse transcription polymerase chain reaction (qRT-PCR) analysis were performed using established protocols. Primer pairs were used as follows: For the sequence of FAM96B, 5’-GGAGTTGAACGTAGTAGAGCAGG-3’ for forward, and 5’-GACAGACCAATAAGGGTGGC-3’ for reverse. For β-actin, the sequences were 5’-TGAGACCTTCAACACCCCAG-3’ and 5’-GCCATCTCTTGCTCGAAGTC- 3’. The PCR reaction system was set to a total volume of 20 µL, comprising 1 × Taq buffer, 0.2 mM dNTPs, 0.5 µM primers, 1.5 mM MgCl_2_, and 1 U of Taq DNA polymerase. The PCR program included an initial denaturation step at 94°C for 5 minutes, followed by 35 cycles, each consisting of 94°C for 30 seconds, 58°C for 30 seconds, and 72°C for 1 minute, culminating in a final extension at 72°C for 10 minutes. The data from qRT-PCR were analyzed using the 2 ^−ΔΔCt^ method.

### Immunohistochemistry and Staining Evaluation

Immunohistochemistry assay was conducted on a total of 137 cases of HCC and paired adjacent non-tumorous samples to assess the protein expression of FAM96B. Tissue samples were fixed in a 4% paraformaldehyde solution, followed by dehydration, clearing, and paraffin embedding. The samples were then cut into thin sections and affixed to glass slides. The sections were deparaffinized using xylene and subsequently rehydrated through a series of decreasing ethanol concentrations. Antigenic sites were exposed through heat repair. Nonspecific binding sites were blocked using goat serum. The primary antibody was added and incubated at 4°C overnight, followed by the addition of the secondary antibody, which was incubated at room temperature for 1 hour. Color development was achieved using DAB. Counterstaining was performed with hematoxylin, after which the sections were dehydrated and mounted with a mounting medium. The primary antibody against FAM96B utilized was rabbit monoclonal antibody (1 : 100; Abcam, Chicago, IL, USA). The expression level of FAM96B was evaluated under double-blinded conditions by analyzing the positivity of cells in all samples for both intensity and extent. Cell staining positivity was classified into 5 groups: score 0 (0%-9%), 1 (10%-25%), 2 (26%-50%), 3 (51%-75%), and 4 (76%-100%). Staining intensity was categorized into 3 levels: 0 (no staining), 1 (low intensity), 2 (moderate intensity), and 3 (high intensity). To determine the cumulative score, ranging from 0 to 12, the product of intensity and extent scores was calculated.

### Public Data

We analyzed the mRNA expression of FAM96B by utilizing RNA sequencing data from 50 HCC cases and their corresponding noncancerous samples retrieved from The Cancer Genome Atlas (TCGA) database (https://cancergenome.nih.gov/).

### Cell Culture

The HepG2 human HCC cell line was acquired from American Type Culture Collection (ATCC) (located in Manassas, VA, USA) and cultured regularly in RPMI-1640 medium (manufactured by Gibco) containing 10% serum at 37°C and 5% CO_2_. Additionally, 100 U/mL penicillin and 100 μg/mL streptomycin were added to the culture medium.

### Western Blot

For Western blot analysis, SDS-PAGE gel was used for electrophoresis, and the proteins in the gel were subsequently transferred to a polyvinylidene fluoride (PVDF) membrane. Block non-specific binding sites on the membrane with 5% skimmed milk powder and incubate for 1 hour at room temperature. Incubate the membrane with a primary antibody against FAM96B (1 : 1000; Abcam, Chicago, IL, USA) overnight at 4°C. Wash the membrane multiple times with tris-buffered saline (TBST) to remove unbound primary antibody. Incubate the membrane with an (Horseradish Peroxidase, HRP)-labeled secondary antibody for 1 hour at room temperature. Add (enhanced chemiluminescence, ECL) chemiluminescence reagent after washing the membrane multiple times with TBST, and use a gel imaging system to detect the luminescence signal to reveal the target protein band.

### Statistical Analysis

All statistical analyses were performed using Statistical Package for Social Sciences (SPSS) Statistics 26.0 software (IBM SPSS Corp.; Armonk, NY, USA). The *χ*^2^ test was applied to analyze the correlations between FAM96B expression and clinicopathologic variables. To compare the mRNA and protein levels of FAM96B in cancerous versus paired noncancerous samples, a paired *t*-test was employed. Survival curves were generated through the Kaplan–Meier method, and differences were evaluated with the log-rank test. Factors potentially affecting survival were identified using Cox’s proportional hazards regression model, incorporating both univariate and multivariate analyses. Significance was attributed to a *P* value of less than .05.

## Results

### Characteristics of Patients

Table 1 provides a detailed summary of the clinical characteristics of the 137 patients diagnosed with HCC. The study population included 78 male and 59 female patients, with a median age of 56 years, ranging from 32 to 75 years. Within this cohort, 69 patients exhibited alpha-fetoprotein (AFP) positivity, 97 tested positive for hepatitis B surface antigen, and 72 presented with BCLC-A stage malignancies.

### FAM96B mRNA Expression Was Decreased in HCC Tissues

**Figure 1.**
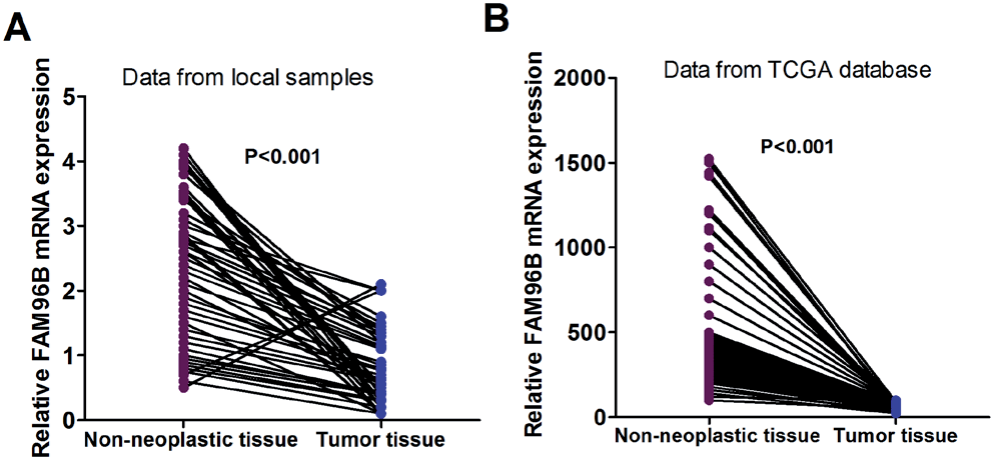
Decreased FAM96B mRNA level in HCC tissues. (A) Relative mRNA expression levels in 137 paired HCC and noncancerous tissues determined by real-time PCR in our study. (B) mRNA expressions validated in The Cancer Genome Atlas (TCGA) database.

The quantification of FAM96B mRNA transcriptional levels in human HCC tissues and their corresponding noncancerous tissues was carried out using quantitative real-time reverse transcription. The findings showed a significant decrease in FAM96B mRNA in liver cancerous tissues compared to noncancerous tissues (*P* < .001, Figure 1A). Additionally, this decrease in FAM96B mRNA expression was further validated by RNAseq data (RNASeqV2) collected from the TCGA database, which also demonstrated lower expression of FAM96B mRNA in tumors (*P* < .001, Figure 1B).

### Immunohistochemistry Analysis of FAM96B Expression in HCC and Its Association with Clinicopathologic Characteristics

**Figure 2.**
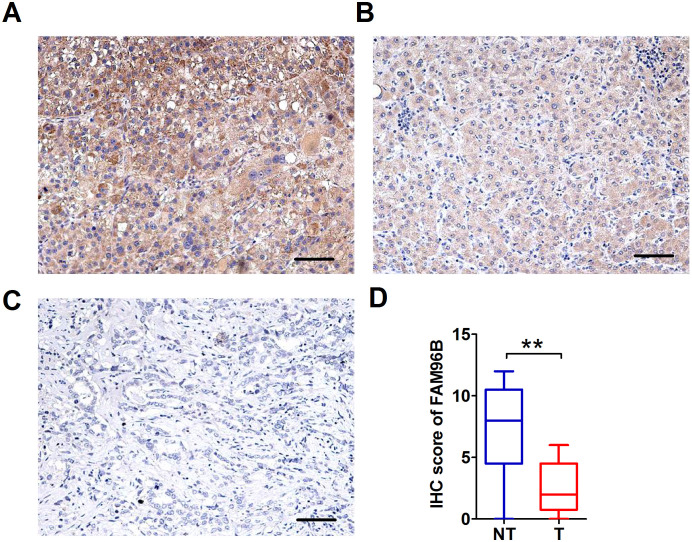
FAM96B expression in HCC tissues by IHC staining. (A) Intensive FAM96B staining in tumor tissues. (B) Moderate FAM96B staining in tumor tissues. (C) Negative FAM96B staining in tumor tissues. (D) IHC score of FAM96B in tumorous tissues and nontumorous tissues. T, tumorous tissues; NT, nontumorous tissues. Scale bar: 50 μm. **P < .01.

In order to confirm the results of real-time PCR, we conducted IHC staining to analyze the expression of FAM96B among 137 HCC samples. Our findings demonstrated varying levels of FAM96B expression in tumor tissues, which were classified as strong positive, weak positive, or negative (Figure 2A-C). Additionally, our observations indicate an increased prevalence of FAM96B-negative cell populations in liver tumor tissues compared with non-tumor liver tissues. Moreover, a decreased immunohistochemical FAM96B score was observed in HCC samples in comparison to nearby noncancerous tissues (*P* < .001) (Figure 2D). The outcomes of the *χ*^2^ examination showed a notable link between inadequate FAM96B levels and tumor size (*P* = .014). Furthermore, a strong relationship was identified between FAM96B levels and BCLC stage (*P* < .001), Child stage (*P* = .003), as well as distant metastasis (*P* = .032). However, no significant correlation with age, gender, smoking, drinking, hepatitis B surface antigen (HBsAg) infection, AFP, tumor number, portal vein tumor thrombus, and cirrhosis was observed (Table 1).

### Association of FAM96B Level with OS in HCC Patients

**Figure 3.**
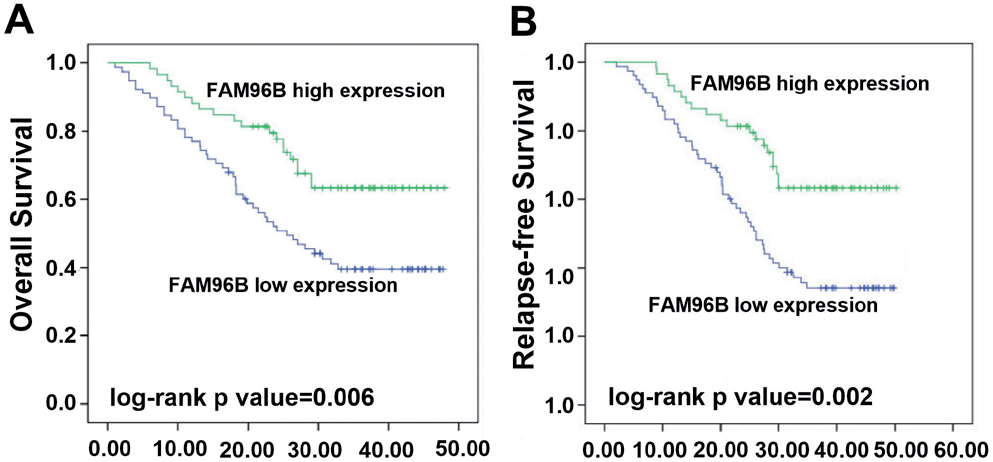
Kaplan–Meier curve analysis of (A) overall survival and (B) disease-free survival in HCC patients by the expression of FAM96B. Log-rank test was used to calculate the difference significance.

Kaplan–Meier curve revealed a significant statistical relationship between low FAM96B expression and poorer OS compared to high FAM96B expression (Figure 3A, log-rank *P* = .006). Based on the results presented in Table 2, the univariate survival analysis indicated a significant association with OS in patients with HCC for various factors. Specifically, these factors include larger tumor size (*P* = .004), elevated BCLC stage (*P* = .002) or higher Child stage (*P* = .024), decreased FAM96B expression (*P* = .002), and the presence of distant metastasis (*P* = .006). Furthermore, multivariate analysis confirmed that low FAM96B expression (HR: 2.05; 95% CI: 1.21-3.44; *P* = .006) was independently associated with OS in individuals with HCC, regardless of age, gender, HBsAg infection, and AFP levels (Table 2).

### Association of FAM96B Level with DFS

Similar to the results on OS, our research also identified a link between FAM96B level and (Disease-free Survival,DFS) in patients. As shown in Figure 3B, our findings indicated that patients with low FAM96B expression exhibited significantly poorer DFS in contrast to those with high FAM96B expression (log-rank *P* = 0.002). Univariate Cox regression analysis demonstrated a significant association between DFS in HCC patients and several factors: larger tumor size (*P* =0 .001), advanced BCLC stage (*P* <0 .001) or Child stage (*P* =0 .042), reduced FAM96B expression (*P* =0 .001), and presence of distant metastasis (*P* = 0.002) (Table 3). Furthermore, our multivariate analysis confirmed that low FAM96B expression independently predicted DFS (HR: 2.24; 95% CI: 1.38-3.68; *P* = 0.001) (Table 3).

### Overexpression of FAM96B Promotes Apoptosis and Inhibits Growth of Liver Cancer Cell

**Figure 4.**
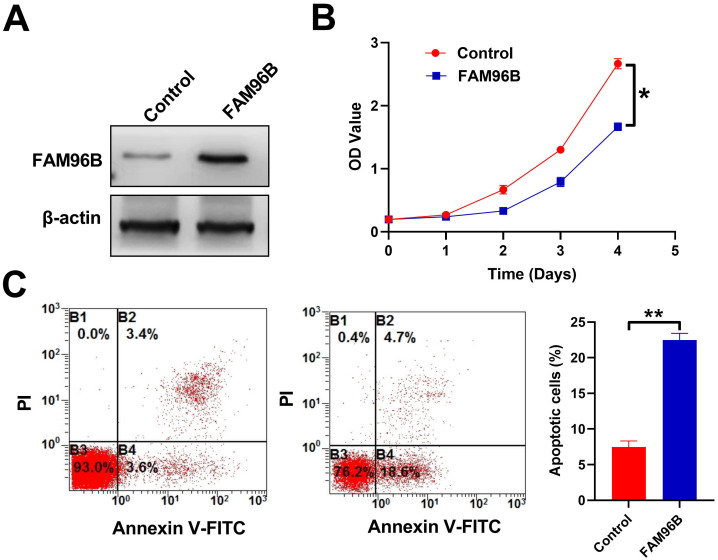
Overexpression of FAM96B promotes apoptosis and inhibits the growth of liver cancer cells. (A) FAM96B expression in HepG2 cells, which was transfected with an expression vector encoding FAM96B. (B) Cell growth curves with FAM96B overexpression. (C) Overexpression of FAM96B induces apoptosis of HepG2 cells. Q1: Mechanically damaged cells; Q2: late apoptotic or necrotic cells; Q3: normal cells; Q4: early apoptotic cells. **P <0 .01.

Our research established a cell line with overexpression of FAM96B, which was confirmed through Western blot analysis (Figure 4A). The findings indicate that FAM96B overexpression hinders the growth of liver cancer cells (Figure 4B) and enhances apoptosis (Figure 4C), suggesting its potential as a tumor suppressor.

## Discussion

Hepatocellular carcinoma is a common type of cancer worldwide. Despite significant advancements in diagnosis and combined therapy over recent years, the prognosis for patients continues to be bleak. Research indicates that the prognosis for patients with HCC remains poor, with the survival rate over a 5-year period remaining less than 20%.^19^ Given the high mortality rate and poor treatment response associated with HCC, identifying new biomarkers implicated in the oncogenesis and progression of the disease would greatly benefit its diagnosis and treatment.^20^ These biomarkers could potentially lead to more effective management strategies.

Recent research has primarily focused on the function of FAM96B in maintaining normal cellular function. Specifically, FAM96B has been implicated in regulating endothelial cell proliferation, migration, and microtubule formation. It has been reported that the helix-loop-helix protein E2-2 is pivotal for maintaining the quiescence of endothelial cells. Yang et al^12^ discovered that FAM96B regulates E2-2 protein levels, thereby interfering with its effects on endothelial cells. A recent study has proposed that FAM96B has the potential to interact with prelamin A and potentially influence cell senescence. This is significant because the harmful buildup of prelamin A can contribute to age-related cell dysfunction.^13^ However, the involvement of FAM96B in HCC is still largely unexplored with limited evidence available.

To explore the protein level of FAM96B and its relationship with clinical parameters and prognosis in patients with HCC, we conducted this research. Our results showed a notable upregulation of FAM96B in HCC specimens relative to non-tumorous ones. Additionally, statistical analysis revealed a significant link between low FAM96B expression in HCC and larger tumor size, higher BCLC or Child stage, and distant metastasis. It appears that FAM96B could potentially play a role as a tumor suppressor in HCC. Patients with low FAM96B levels exhibit a negative impact on DFS and OS. Additionally, our analysis revealed that FAM96B serves as an independent predictor of OS and DFS, as observed in univariate and multivariate survival analyses. The results of this study showed that low expression of FAM96B is closely related to the poor prognosis of HCC patients, which is consistent with previous research results on the role of FAM96B in breast cancer.^18^ Our experiments also further revealed that FAM96B inhibits HCC progression by promoting apoptosis and inhibiting the proliferation of HCC cells. These findings not only enrich our understanding of the function of FAM96B, but also provide new ideas for the treatment of HCC. Our results also indicated that low expression of FAM96B could be used as an independent indicator to predict the prognosis of HCC patients. This finding has important clinical implications. The expression level of FAM96B can be used to assess a patient’s prognostic risk and help doctors develop more targeted treatment plans. In addition, by monitoring the dynamic changes of FAM96B, tumor recurrence or progression may be detected at an early stage, and treatment strategies can be adjusted in a timely manner. Collectively, these insights propose that FAM96B inactivation could be an important event in the development of HCC. Hence, this study holds clinical significance and practical value.

However, the precise process through which FAM96B hinders the progression of HCC is not yet fully understood. In addition to our study finding that FAM96B inhibits tumor progression by inducing tumor cell apoptosis and inhibiting their growth in HCC, FAM96B may also exert its anti-cancer effects through other pathways. Although in breast cancer research, FAM96B has been reported to inhibit cancer progression by regulating the Wnt/β-catenin signaling pathway,^18^ its specific mechanism of action in HCC remains unclear. FAM96B forms an integral component of the MMXD complex (MMS19-FAM96B-XPD) and plays a critical role in chromosome segregation as well as the creation of the mitotic spindle.^15^ The downregulation of FAM96B in HCC may disrupt mitosis and result in increased accumulation of heterotypic nuclei, thus promoting tumor progression. Additionally, FAM96B is critical for the assembly of cytosolic iron-sulfur (Fe–S) clusters in specific Fe–S proteins that participate in DNA metabolism, which is vital for DNA repair and genomic stability.^14^ Therefore, the downregulation of FAM96B may contribute to the progression of HCC by impacting DNA damage repair. Our study reveals that FAM96B inhibits HCC progression via affecting the growth and cell death of tumor cells. Overexpression of FAM96B induces apoptosis in HepG2 cells and inhibits their growth. Both previous reports and our findings suggest that FAM96B can influence the progression of HCCthrough various mechanisms. However, the specific mechanisms by which FAM96B exerts its anti-cancer effects in HCC remain obscure and warrant additional investigative efforts.

In summary, our investigation revealed that there was a decrease in FAM96B levels in human HCC and it exhibits a significant association with tumor cell growth and apoptosis. Furthermore, we found that FAM96B was independently linked to both the OS and DFS in individuals with HCC. These results indicate that FAM96B may function as a promising indicator for predicting tumor relapse and monitoring patient outcomes in HCC, holding some clinical significance and practical value.

## Figures and Tables

**Table 1.** Relationship Between Tumor FAM96B Expression and Clinic Features

Variables	No. of Cases	FAM96B Expression		*P*
		Low	High	
All	137	78	59	
Age				.343
≤50	38	19	19	
>50	99	59	40	
Gender				.161
Female	59	38	21	
Male	78	40	38	
Smoking				.226
Yes	69	43	26	
No	68	35	33	
Alcohol				.487
Yes	66	40	26	
No	71	38	33	
HBsAg infection				.456
Yes	97	53	44	
No	40	25	15	
AFP (ng/mL)				.862
>200	69	40	29	
≤200	68	38	30	
Tumor size (cm)				**.014**
>5	79	52	27	
≤5	58	26	32	
Tumor number				.861
Solitary	75	42	33	
Multiple	62	36	26	
BCLC stage				<** .001**
A stage	72	23	49	
B + C stage	65	55	10	
Child stage				**.003**
A stage	76	35	41	
B stage	61	43	18	
Distant metastasis Yes No PVTT Yes No Cirrhosis Yes No	56 81 57 80 66 71	38 40 34 44 39 39	18 41 23 36 27 32	**.032** .611 .733

*P* value when expression levels were compared using the Pearson *χ*2 test, and *P* value < .05 was considered statistically significant..

AFP, alpha fetoprotein; BCLC, Barcelona clinic liver cancer; HBsAg, hepatitis B surface antigen; PVTT, portal vein tumor thrombus..

**Table 2.** Association of FAM96B and Clinical Factors with Overall Survival

	Unadjusted HR^*^ (95% CI)	*P*	Adjusted HR^†^ (95% CI)	*P*
FAM96B low expression	2.06 (1.23-3.37)	**0.002**	2.05 (1.21-3.44)	**0.006**
Gender	0.62 (0.36-1.06)	0.090	–	–
Age at diagnosis	0.77 (0.48-1.26)	0.278	–	–
Smoking	0.99 (0.64-1.61)	0.94	–	–
Alcohol	1.11 (0.67-1.81)	0.62	–	–
HBsAg infection	0.87 (0.48-1.51)	.64	–	–
AFP (ng/mL) Tumor size (cm) Tumor number BCLC stage Child stage Distant metastasis PVTT Cirrhosis	1.13 (0.69-1.81) 2.31 (1.35-3.89) 0.82 (0.50-1.35) 2.42 (1.41-4.25) 1.79 (1.10-2.91) 2.16 (1.28-3.62) 0.94 (0.58-1.57) 0.91 (0.54-1.51)	0.72 **0.004** 0.55 **0.002** **0.024** **0.006** 0.97 0.83	– 1.94 (1.13-3.35) 4.6 1(1.44-14.52) 1.38 (0.83-2.29) 0.43 (0.12-1.41) – –	– **0.022** **0.0009** 0.253 0.212 – –

AFP, alpha fetoprotein; BCLC, Barcelona clinic liver cancer; CI, confidence interval; HBsAg, hepatitis B surface antigen; HR, hazard ratio; PVTT, portal vein tumor thrombus.

^*^Hazard ratios in univariate models.

^†^Hazard ratios in multivariable models.

**Table 3.** Association of FAM96B and Clinical Factors with Relapse-Free Survival

	Unadjusted HR^*^ (95% CI)	*P*	Adjusted HR^†^ (95% CI)	*P*
FAM96B low expression	2.12 (1.31-3.42)	**0.001**	2.24 (1.38-3.68)	**0.001**
Gender	0.77 (0.42-1.14)	0.227	–	–
Age at diagnosis	0.69 (0.42-1.11)	0.098	–	–
Smoking	1.08 (0.64-1.72)	0.631	–	–
Alcohol	1.23 (0.73-2.02)	0.282	–	–
HBsAg infection	0.96 (0.55-1.58)	0.732	–	–
AFP (ng/mL) Tumor size (cm) Tumor number BCLC stage Child stage Distant metastasis PVTT Cirrhosis	1.02 (0.62-1.71) 2.26 (1.31-3.83) 0.89 (0.57-1.42) 2.72 (1.58-4.69) 1.61 (1.01-2.58) 2.45 (1.48-4.12) 1.06 (0.66-1.69) 0.92 (0.56-1.48)	0.75 **0.001** 0.537 **<0 .001** **0.042** **0.002** 0.969 0.831	– 1.91 (1.12-3.32) 5.19 (1.63-16.49) 1.31 (0.82-2.09) 0.54 (0.24-1.62) – –	– **0.015** **0.008** 0.39 0.242 – –

AFP, alpha fetoprotein; BCLC, Barcelona clinic liver cancer; CI, confidence interval; HBsAg, hepatitis B surface antigen; HR, hazard ratio; PVTT, portal vein tumor thrombus.

^*^Hazard ratios in univariate models.

^†^Hazard ratios in multivariable models.

## Data Availability

The data that support the findings of this study are available on request from the corresponding author.

## References

[1] Sung H Ferlay J Siegel RL , et al. Global cancer statistics 2020: GLOBOCAN estimates of incidence and mortality worldwide for 36 cancers in 185 countries. CA Cancer J Clin. 2021;71(3):209 249. (10.3322/caac.21660) 33538338

[2] Kollar J Drizdal T Vrba J , et al. Microwave catheter navigation system for the radiofrequency liver ablation. Cancers (Basel). 2022;14(21):5296. (10.3390/cancers14215296) 36358714 PMC9656965

[3] Zhang K Wang T Zhou H , et al. A novel Aurora-A inhibitor (MLN8237) synergistically enhances the antitumor activity of sorafenib in hepatocellular carcinoma. Mol Ther Nucleic Acids. 2018;13:176 188. (10.1016/j.omtn.2018.08.014) 30292139 PMC6172479

[4] You X Jiang W Lu W , et al. Metabolic reprogramming and redox adaptation in sorafenib-resistant leukemia cells: detected by untargeted metabolomics and stable isotope tracing analysis. Cancer Commun (Lond). 2019;39(1):17. (10.1186/s40880-019-0362-z) 30947742 PMC6449955

[5] Dileepan M Ge XN Bastan I , et al. Regulation of eosinophil recruitment and allergic airway inflammation by tropomyosin receptor kinase A. J Immunol. 2020;204(3):682 693. (10.4049/jimmunol.1900786) 31871023 PMC7058110

[6] Ridder DA Urbansky LL Witzel HR , et al. Transforming growth factor-beta activated kinase 1 (Tak1) is activated in hepatocellular carcinoma, Mediates Tumor Progression, and Predicts Unfavorable Outcome. Cancers(Basel). 2022;14(2):430. (10.3390/cancers14020430) 35053591 PMC8774263

[7] Ghouri YA Mian I Rowe JH . Review of hepatocellular carcinoma: epidemiology, etiology, and carcinogenesis. J Carcinog. 2017;16:1. (10.4103/jcar.JCar_9_16) 28694740 PMC5490340

[8] Costentin CE Bababekov YJ Zhu AX Yeh H . Is it time to reconsider the Milan criteria for selecting patients with hepatocellular carcinoma for deceased-donor liver transplantation? Hepatology. 2019;69(3):1324 1336. (10.1002/hep.30278) 30229978

[9] Sayiner M Golabi P Younossi ZM . Disease burden of hepatocellular carcinoma: a global perspective. Dig Dis Sci. 2019;64(4):910 917. (10.1007/s10620-019-05537-2) 30835028

[10] Karaoğullarından Ü Üsküdar O Odabaş E Ak N Kuran S . Hepatocellular carcinoma in cirrhotic versus noncirrhotic livers: clinicomorphologic findings and prognostic factors. Turk J Gastroenterol. 2023;34(3):262 269. (10.5152/tjg.2023.21791) 36688381 PMC10152162

[11] Hu T Li J Zhang C , et al. The potential value of microRNA-4463 in the prognosis evaluation in hepatocellular carcinoma. Genes Dis. 2017;4(2):116 122. (10.1016/j.gendis.2017.03.003) 30258914 PMC6136594

[12] Yang W Itoh F Ohya H , et al. Interference of E2-2-mediated effect in endothelial cells by FAM96B through its limited expression of E2-2. Cancer Sci. 2011;102(10):1808 1814. (10.1111/j.1349-7006.2011.02022.x) 21722264

[13] Xiong XD Wang J Zheng H , et al. Identification of FAM96B as a novel prelamin A binding partner. Biochem Biophys Res Commun. 2013;440(1):20 24. (10.1016/j.bbrc.2013.08.099) 24041693

[14] Stehling O Vashisht AA Mascarenhas J , et al. MMS19 assembles iron-sulfur proteins required for DNA metabolism and genomic integrity. Science. 2012;337(6091):195 199. (10.1126/science.1219723) 22678362 PMC3420340

[15] Ito S Tan LJ Andoh D , et al. MMXD, a TFIIH-independent XPD-MMS19 protein complex involved in chromosome segregation. Mol Cell. 2010;39(4):632 640. (10.1016/j.molcel.2010.07.029) 20797633

[16] Zhang XH Chen XJ Yan YB . Fam96b recruits brain-type creatine kinase to fuel mitotic spindle formation. Biochim Biophys Acta Mol Cell Res. 2023;1870(2):119410. (10.1016/j.bbamcr.2022.119410) 36503010

[17] Liang H Li W Yang H , et al. FAM96B inhibits the senescence of dental pulp stem cells. Cell Biol Int. 2020;44(5):1193 1203. (10.1002/cbin.11319) 32039527

[18] Zhang DD Sun XL Liang ZY Wang XY Zhang LN . FAM96A and FAM96B function as new tumor suppressor genes in breast cancer through regulation of the Wnt/beta-catenin signaling pathway. Life Sci. 2022;308:120983. (10.1016/j.lfs.2022.120983) 36165859

[19] Chen MY Juengpanich S Hu JH , et al. Prognostic factors and predictors of postoperative adjuvant transcatheter arterial chemoembolization benefit in patients with resected hepatocellular carcinoma. World J Gastroenterol. 2020;26(10):1042 1055. (10.3748/wjg.v26.i10.1042) 32205995 PMC7081004

[20] Wu X Wan R Ren L Yang Y Ding Y Wang W . Circulating microRNA panel as a diagnostic marker for hepatocellular carcinoma. Turk J Gastroenterol. 2022;33(10):844 851. (10.5152/tjg.2022.21183) 35943150 PMC9623203

